# Colonic epithelial ion transport is not affected in patients with diverticulosis

**DOI:** 10.1186/1471-230X-7-37

**Published:** 2007-09-23

**Authors:** Philip S Osbak, Niels Bindslev, Steen S Poulsen, Nicolai Kaltoft, Maria C Tilotta, Mark B Hansen

**Affiliations:** 1 Department of Gastrointestinal Surgery K, Bispebjerg University Hospital of Copenhagen, Denmark; 2 Department of Medical Physiology, Panum Institute, University of Copenhagen, Denmark; 3 Department of Anatomy B, Panum Institute, University of Copenhagen, Denmark

## Abstract

**Background:**

Colonic diverticular disease is a bothersome condition with an unresolved pathogenesis. It is unknown whether a neuroepithelial dysfunction is present. The aim of the study was two-fold; (1) to investigate colonic epithelial ion transport in patients with diverticulosis and (2) to adapt a miniaturized Modified Ussing Air-Suction (MUAS) chamber for colonic endoscopic biopsies.

**Methods:**

Biopsies were obtained from the sigmoid part of the colon. 86 patients were included. All patients were referred for colonoscopy on suspicion of neoplasia and they were without pathological findings at colonoscopy (controls) except for diverticulosis in 22 (D-patients). Biopsies were mounted in MUAS chambers with an exposed area of 5 mm^2^. Electrical responses to various stimulators and inhibitors of ion transport were investigated together with histological examination. The MUAS chamber was easy to use and reproducible data were obtained.

**Results:**

Median basal short circuit current (SCC) was 43.8 μA·cm^-2 ^(0.8 – 199) for controls and 59.3 μA·cm^-2 ^(3.0 – 177.2) for D-patients. Slope conductance was 77.0 mS·cm^-2 ^(18.6 – 204.0) equal to 13 Ω·cm^2 ^for controls and 96.6 mS·cm^-2 ^(8.4 – 191.4) equal to 10.3 Ω·cm^2 ^for D-patients. Stimulation with serotonin, theophylline, forskolin and carbachol induced increases in SCC in a range of 4.9 – 18.6 μA·cm^-2^, while inhibition with indomethacin, bumetanide, ouabain and amiloride decreased SCC in a range of 6.5 – 27.4 μA·cm^-2^, and all with no significant differences between controls and D-patients. Histological examinations showed intact epithelium and lamina propria before and after mounting for both types of patients.

**Conclusion:**

We conclude that epithelial ion transport is not significantly altered in patients with diverticulosis and that the MUAS chamber can be adapted for studies of human colonic endoscopic biopsies.

## Background

Colonic diverticular disease is a bothersome condition for both patient and clinician and difficult to treat. In Western countries the prevalence of diverticulosis is 5 % of individuals under 40 years of age but increases to as high as 65 % of individuals aged 65 or more [1]. The most frequently reported symptoms of diverticular disease are constipation, diarrhoea, pain, and bleeding.

The pathogenesis of diverticulosis and its structural and functional components are still unresolved. Diverticulosis has been epidemiologically [2] and functionally related to diet poor in fibers and to increased intracolonic pressure [3,4]. Therefore alterations in the neuromuscular and neuroepithelial functions have been suggested. Supporting this hypothesis, a recent study in smooth muscle cells from the sigmoid part of the colon in patients with diverticulosis points to a state of neuromuscular dysfunction with hypersensitivity to acetylcholine due to a decreased cholinergic innervation [5].

Functional studies of intestinal epithelial transport in humans are restricted mainly to in vitro methods including everted sacs, intestinal rings, and specimens from surgical [6,7]. and endoscopic proceduces [8] mounted in conventional Ussing chambers. Recently we developed a miniaturized Modified Ussing Air-Suction (MUAS) chamber for the study of human duodenal endoscopic biopsies [9].

We hypothesize that changes in neuroepithelial integrity and epithelial ion transport are present in patients with diverticulosis and that the MUAS chamber can be adapted for the study of epithelial ion transport in human colonic endoscopic biopsies.

To test these hypotheses we investigated biopsies obtained during rutine coloscopy in patients without (controls) and with (D-patients) macroscopically detectable colonic diverticulosis. Electrical parameters and histology were examined in this context.

## Materials and methods

### Study population

The study included endoscopic biopsies from 86 patients of which 41 were women. The median age was 64 years (range 20 – 98). All patients were referred to colonoscopy for examination on suspicion of colonic neoplasia. The included subjects were without pathological findings at colonoscopy (controls) except for diverticulosis in the left part of the colon in 22 cases (D-patients).

### Ethics

Our study-protocol was approved by the Scientific Ethical Committee for Copenhagen (KA 97161) and Frederiksberg Counties (KF01-232/03) and conducted in accordance with the Declaration of Helsinki V. All patients gave written informed consent. All signs of disease in colon were noted. The patients' medication at the time of examination was noted.

### Mounting of biopsies and electrical measurements

Five biopsies were obtained from the sigmoid part of the colon (30 cm aborally to the anus on retraction of the endoscope) using a standard biopsy forceps (Boston Scientific, Denmark). The biopsies were taken from macroscopically normal appearing mucosa, not from the diverticuli per se.

Biopsies were transported in ice-cold bicarbonate-Ringer solution to the laboratory. They were mounted within 30 min in MUAS chambers, which uses constant air suction to fixate biopsies [9]. Mounting was carried out at 10 times magnification by means of a stereomicroscope to secure mucosa-serosa orientation and proper fixation. The exposed tissue area was 5 mm^2^. Both sides of the tissue were bathed with a bicarbonate-Ringer solution containing the following (in mM): 140 Na^+^, 4 K^+^, 121 Cl^-^, 1 Ca^2+^, 0.5 Mg^2+^, 0.5 SO_4_^2-^, and 25 HCO_3_^-^, oxygenated with 95 % O_2_/5 % CO_2_, circulated by gas-lift. Media at the serosal side were further added 11 mM D-glucose and 11 mM D-sorbitol at the mucosal side. Temperature was maintained at 37°C by water jackets. Short-circuit current (SCC) measured in μA·cm^-2 ^and slope conductance (G) in mS·cm^-2 ^were recorded continuously by an automated voltage-clamp device [9]. Correction for the resistance in solutions was performed immediately before each new tissue was mounted.

The height of the suction sleeve in a MUAS chamber is of importance to mounting of tissue. For biopsies to stay in place, we found that the height of the sleeve should be reduced to 40 μm to fit the colon biopsies compared to 50 μm for duodenal biopsies [9].

Experiments were performed after an equilibration period of 15 min. In order to evaluate tissue viability and transport capacity, application of various stimulators (serotonin, 5-hydroxytryptamine, 5-HT; forskolin; theophylline; carbachol) and inhibitors (amiloride; indometacin; bumetanide; ouabain) of epithelial ion transport were added to the serosal bathing solution, except for amiloride which was added to the mucosal solution and for indometacin added to both sides.

### Compounds

All drugs were purchased from Sigma (Vallensbaek Strand, Denmark) except for bumetanide, which was a gift from Leo Pharmaceuticals, Denmark.

## Data and statistical analysis

### Statistical analysis

Data are presented as median and range (minimum-maximum) followed by (N = number of patients, n = number of biopsies). Mann-Whitney rank sum test was used for statstical analysis according to results of normality and equal variance tests (SigmaStat 2.0 for Windows, SPSS Inc., USA). P < 0.05 was considered significant. The effect of compounds was defined as data before compared to data after application of the compounds.

In a supplementary data-sheet we present a vertical point plot of SCC and G for the individual patients, additional file 1.

### Histological examination

Protocols were blinded to the examiner. Fixation was performed in 4 % buffered paraformaldehyde after taking biopsies and after experiments in the MUAS chamber. The fixed tissue samples were then dehydrated and embedded in paraffin and cut into 10 μm sections. Sections were stained with hematoxylin/periodic acid Schiff, examined and photographed using a Leitz Ortoplan microscope (Wetzlar, Germany) fitted with a cooled camera (Evolution MP, MediaCybernetics, Wokingham, Berkshire, UK).

## Results

### Medication and comorbidity

Thirty-seven patients were under current medical treatment for arterial hypertension, 21 for mental or other neurological disorders, 10 for ischaemic heart disease, 9 for asthma or chronic obstructive lung disease, 9 for diabetes mellitus, and 2 for thyroid disease. 37 patients did not take any medication at all and were equally distributed in control and D-patient groups. Median systolic blood pressure was 140 mmHg (range 188 – 98), median diastolic blood pressure was 82 mmHg (range 112 – 72) and median heart rate was 78 (range 48 – 110), with no significant difference between controls and D-patients (p = 0.734, p = 0.805, p = 0.242, respectively). No complications due to endoscopy were reported.

### Electrical parameters

Biopsies were excluded based on unstable SCC and G or on no response to any of the secretagogues. Of 5 biopsies from the same patient at least 3 were viable and (at least) 2 of these 3 gave stable measurements leaving us with a success-rate of about 40 %.

### Basal observations

After an equilibration period in the chamber, basal SCC was 43.8 μA·cm^-2 ^(64, 120) for controls and 59.3 μA·cm^-2 ^(22, 40) for D-patients with no significant difference (p = 0.106), table 1. Results for G are shown as well in table 1 and were also not significantly different. During basal conditions, these parameters remained stable for more than 2 hrs. Furthermore, reproducible SCC-responses could be obtained for up to 2 hrs after mounting for all the tested stimulators and inhibitors of ion transport. In the majority of experiments, a slight progressive increase in SCC and G appeared within the 3 hrs and after 6–8 hrs a more than 100 % increase in SCC and G was observed (data not shown).

**Table 1:** Basal parameters. Short circuit current (SCC) and conductance (G) is shown for controls and D-patients.

		**N, n**	**Median**	**Range**	**P value**	**Mean**	**SEM**
			*μA·cm*^-2^	*μA·cm*^-2^			
Controls	SCC	64, 120	43.8	0.8–199.0		57.1	5.0
D-patients	SCC	22, 40	59.3	3.0–177.2	0.106	63.2	7.0
			*mS·cm*^-2^	*mS·cm*^-2^			
Controls	G	64, 127	77.0	18.6–204.0		84.3	5.0
D-patients	G	21, 46	96.6	8.4–191.4	0.618	101.6	6.8

### Stimulation

#### Effect of secretagogues on SCC

**Figure 1:**
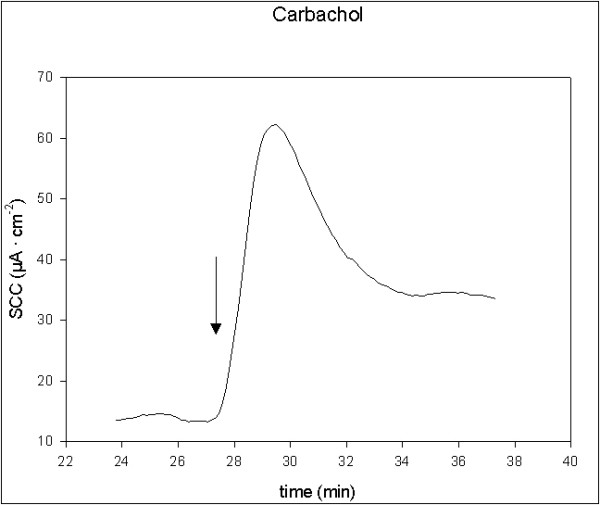
Trace showing the change in SCC following application of carbachol, 10 μM (N = 6, n = 18). Arrow marks the time of adding the compoud.

**Figure 2:**
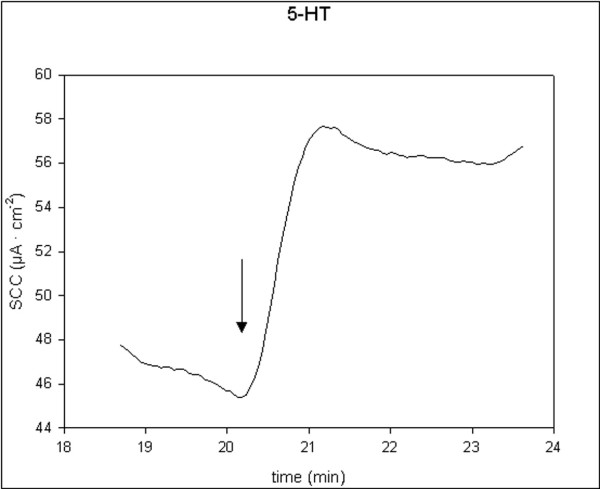
Trace showing the change in SCC following application of 5-HT, 100 μM (N = 21, n = 29). Arrow marks the time of adding the compoud.

**Figure 3:**
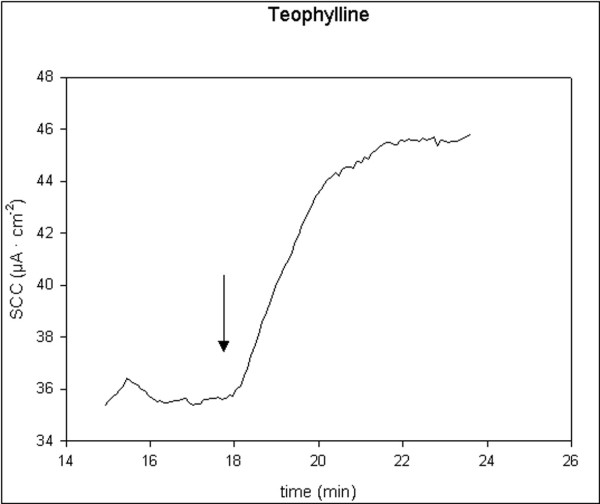
Trace showing the change in SCC following application of theophylline 100 μM (N = 31, n = 39). Arrow marks the time of adding the compoud.

**Figure 4:**
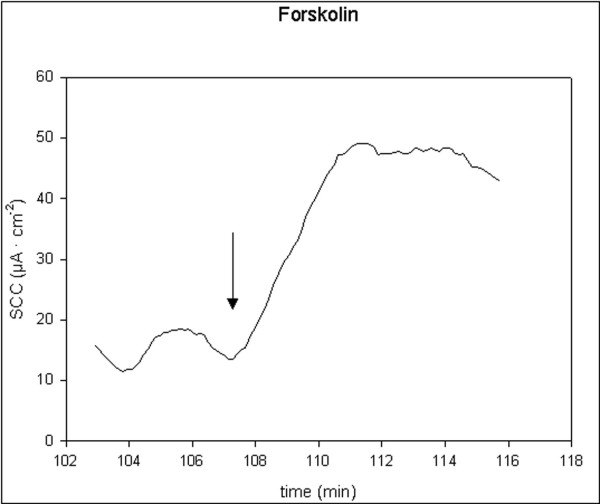
Trace showing the change in SCC following application of forskolin, 1 μM (N = 23, n = 32). Arrow marks the time of adding the compoud.

All applied stimulators of ion secretion induced a significant increase in SCC. 5-HT and theophylline induced rapid, transient small increases, t_1/2 _< 2 min. Carbachol induced rapid, transient and moderate increases, t_1/2 _< 2 min, while forskolin induced prolonged and moderate increases, t_1/2 _> 2 min. Single typical examples of these effects are shown in figures 1, 2, 3, 4. There were no significant differences in responses to all secretagogues between controls and D-patients, table 2.

**Table 2:** Stimulation. Increases in short circuit current (SCC) after stimulation with 5-HT (100 μM), forskolin (1 μM), theophylline (100 μM) and carbachol (10 μM).

	**Compound**	**N, n**	**Median**	**Range**	**P value**	**Mean**	**SEM**
			*μA·cm*^-2^	*μA·cm*^-2^			
Controls	5-HT	12, 15	6.2	0.2–45.6		9.9	3.2
D-patients	5-HT	9, 14	4.9	0.2–20.2	0.807	7.2	1.7
Controls	Forskolin	14, 19	18.6	0.1–109.0		26.8	7.0
D-patients	Forskolin	9, 13	14.0	0.7–60.0	0.619	16.5	5.6
Controls	Theophylline	20, 26	7.3	0.1–62.6		12.9	3.5
D-patients	Theophylline	11, 13	7.8	0.3–36.8	0.572	12.9	3.1
Controls	Carbachol	3, 9	12.2	4.8–46.6		18.1	5.7
D-patients	Carbachol	3, 9	11.2	4.4–64.4	0.508	25.4	9.2

#### Effects of secretagogues on G

In general, SCC-increases in response to stimulators larger than 20 μA·cm^-2 ^were accompanied by an initial increase in G within about 2 min, followed by a decrease within the next 5 – 10 min. When induced by 5-HT or theophylline, the decreasing phase overshooted the initial resting level resulting in an absolute decrease in G. The theophylline-induced increase in G was considerably larger than those induced by either 5-HT or forskolin, and the following decrease did not overshoot the initial resting level. When induced by carbachol there was only a smaller increase in G and the following decrease did not overshoot the resting level. For SCC-changes less than 20 μA·cm^-2 ^there were either only marginal or no measurable changes in G.

### Inhibition

#### Effect of inhibitors on SCC

**Figure 5:**
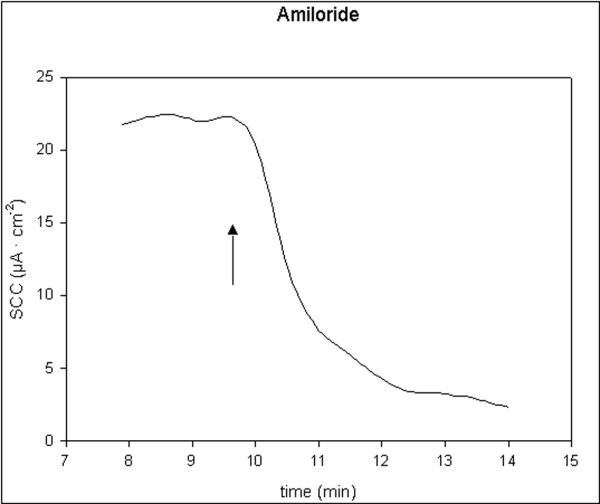
Trace showing the change in SCC following application of amiloride, 100 μM (N = 20, n = 43). Arrow marks the time of adding the compoud.

**Figure 6:**
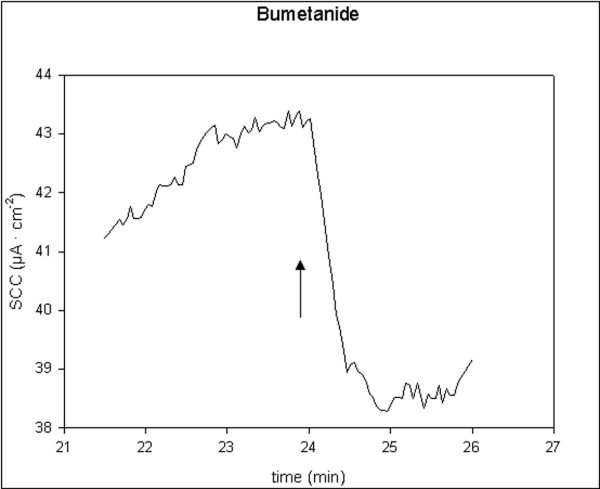
Trace showing the change in SCC following application of bumetanide, 2.5 μM (N = 16, n = 26). Arrow marks the time of adding the compoud.

**Figure 7:**
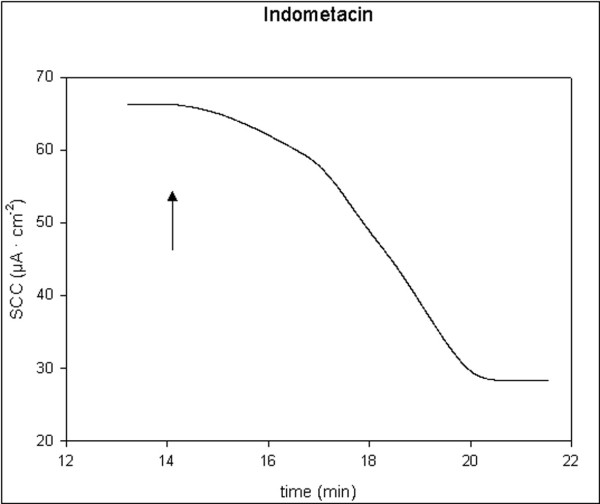
Trace showing the change in SCC following application of indometacin, 40 μM (N = 17, n = 27). Arrow marks the time of adding the compoud.

**Figure 8:**
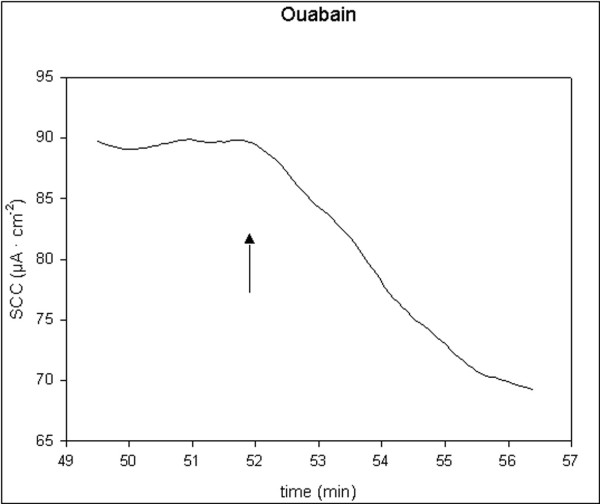
Trace showing the change in SCC following application of ouabain, 200 nM (N = 28, n = 43). Arrow marks the time of adding the compoud.

All applied inhibitors of ion secretion induced long-lasting decreases in SCC. Indometacin and bumetanide induced fast moderate decreases, t_1/2 _< 2 min. Ouabain induced slow and large decreases, t_1/2 _> 3 min. Amiloride induced fast and small decreases, t_1/2 _< 1 min. Single typical examples of these effects are shown in figures 5, 6, 7, 8. For all four drugs, there were no significant differences in responses to all inhibitors between controls and D-patients, table 3.

**Table 3:** Inhibition. Decreases in short circuit current (SCC) after inhibition with indometacin (40 μM), bumetanide (2.5 μM), ouabain (200 nM) and amiloride (100 μM).

	**Compound**	**N, n**	**Median**	**Range**	**P value**	**Mean**	**SEM**
			*μA·cm*^-2^	*μA·cm*^-2^			
Controls	Indometacin	14, 20	16.0	3.4–41.4		11.2	4.3
D-patients	Indometacin	3, 7	8.6	6.6–17.4	0.112	7.7	2.5
Controls	Bumetanide	12, 18	19.0	3.6–57.0		20.0	3.8
D-patients	Bumetanide	4, 8	18.9	9.6–38.5	0.475	20.2	4.5
Controls	Ouabain	20, 31	27.4	4.0–103.4		33.9	5.5
D-patients	Ouabain	8, 12	22.0	2.8–34.0	0.394	20.6	3.3
Controls	Amiloride	16, 28	6.5	2.0–40.0		11.9	2.1
D-patients	Amiloride	4, 15	8.0	1.6–27.4	0.475	8.8	1.9

#### Effects of inhibitors on G

In general, SCC-decreases in response to inhibitors larger than 20 μA·cm^-2 ^were accompanied by a decrease in G within about 2 min, followed by a return to the resting level within 5 – 10 min. When induced by indometacin or bumetanide, the recovery phase did not overshoot the initial resting level resulting in an absolute decrease in G. Indometacin-induced decrease in G was considerably larger than those induced by amiloride, bumetanide and ouabain. When induced by ouabain there was only a smaller decrease in G with a magnitude of 4 mS·cm^-2^. The following increase did not differ from the resting level. For SCC-changes less than 20 μA·cm^-2 ^there were either only marginal or no measurable changes in G.

### Histological examination

**Figure 9:**
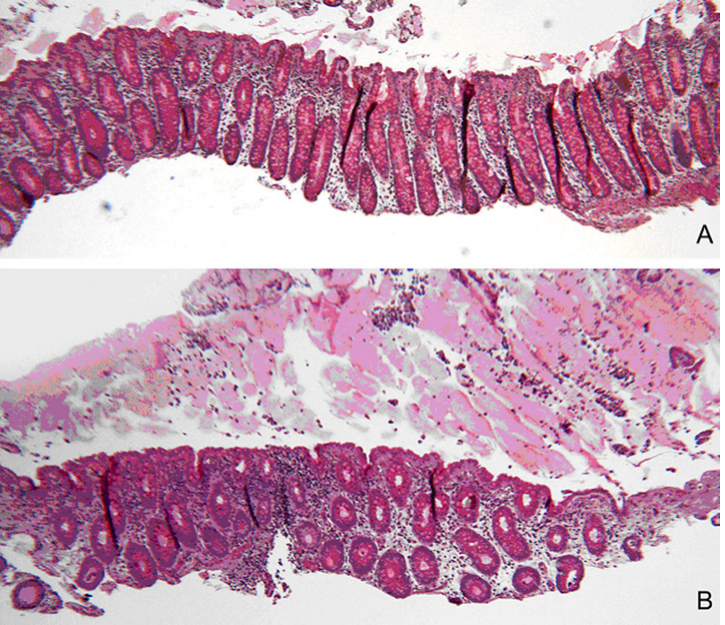
Micrographs of hematoxylin/periodic acid Schiff stained colonic biopsies. (A) biopsy from control patient – not mounted. (B) biopsy from control patient – mounted for 2 hrs in the MUAS chamber. The histology is the same and in particular the epithelial surface is intact. Similar results exist for biopsies from D-patients, not shown.

Histological assessments were performed for the extent of tissue and edge damage and the thickness of biopsies. The damage found in each biopsy was denoted by a severity score, as previously described [10]. When examined in the stereomicroscope, it was noticed that specimens exposed in the MUAS chamber as compared with controls in most cases showed very little tissue damage. The damage seemed to originate from the biopsy forceps more than the chamber, because there was no difference in epithelial damage in the experimental biopsies in comparison with the control biopsies apart from possible minor indications of edge damage. The lack of damage to the surface epithelium following MUAS chamber exposure was confirmed at histological examination. The depth of biopsies varied somewhat and always included the surface epithelium and entire lamina propria. Several biopsies also included the lamina muscularis mucosae and some had parts of the submucosal layer preserved. Two examples without lamina muscularis are shown in figure 9. No difference in histology was detectable for D-patients as compared to controls. In particular, no signs of inflammation were detected in the biopsies from D-patients. When basal electrical parameters were compared with the extent of epithelial damage found in the biopsies on histological examination, there was no clear correlation between the different epithelial damage scores from biopsies scored 0 or 1 vs. biopsies scored 2 or 3.

## Discussion

This study demonstrates that basic and stimulated ion transport does not seem to be altered in patients with diverticulosis and that the MUAS chamber can be adapted for the study of epithelial ion (electrogenic) transport in endoscopic biopsy specimens from the human colon.

### Diverticulosis of the colon

The study provides information about the electropathophysiological characteristics of diverticulosis in the sigmoid part of the colon. We hypothesized the existence of changes in epithelial ion transport functions in D-patients similar to those found in smooth muscle cells. However, this study did not demonstrate such changes, suggesting that epithelial ion transport and probably the neuroepithelial integrity is not altered in patients with diverculosis, table 1, 2, and 3.

The etiology of colonic diverticulosis is unknown. It is assumed that a diet low on fibres is related to colonic diverticulosis. However, there does not seem to be a clear relation between increased intracolonic pressure and the appearance of divertculosis in the sigmoid part of the colon [11]. A recent study suggested an altered neuromuscular function as patients with diverticulosis demonstrated a decrease in acetylcholine transferase activity, an up-regulation of muscarinic M_3 _receptors and an increase in reactivity to exogenous acetylcholine [5] in smooth muscle cells of the sigmoid part of the colon. This hypersensitivity to acetylcholine could result from decreased cholinergic innervation [5]. In the present study there is no indication of such an altered muscarinic receptor sensitivity, table 2.

### The MUAS chamber

The Ussing chamber technique is widely used to characterize epithelial ion transport in the gut. The conventional Ussing chamber has been of limited use for human studies because of limited availability of tissue samples of adequate size, i.e. surgical specimens. Endoscopically obtained tissue samples are desirable due to their wide availability and the tissues are probably less physiologically altered from its original conditions because of less surgical stress and shorter time at ambient temperature than in surgical specimens, that are often without perfusion for some time before the tissue is placed in cold bicarbonate-Ringer. Biopsies are more readily available for a wider array of diseases. Furthermore the patients can be matched with healthy controls not undergoing surgery or even healthy volunteers. Accordingly, the Ussing chamber technique has been refined for the study of small specimens obtained during endoscopy using capsule systems or large size biopsy forceps [9,12-16]. In human biopsy studies the epithelial area in different chambers usually varies from 3 to 5 mm^2 ^[12-14], but ranges from 1 mm^2 ^[17] up to 13.2 mm^2^[15].

Reproducible measurements require a high sensitivity of the equipment and great care in the phase of mounting. Various principles of mounting biopsies in the modified Ussing chambers have been tried. The use of mucosal discs, gluing of specimens, and placement on filter paper has been employed for fixation of specimens. In worry these techniques could cause significant mechanical or chemical edge damage, increase the thickness of unstirred layers and ultimately interfere with the function.

The MUAS chamber has been developed and evaluated for the duodenum for use with (human) forceps for small biopsy specimens [9]. The MUAS chamber has proven its value in functional characterization of muscarinic, prostanoid and serotonin receptors in human duodenum [10,18,19]. The present study suggests that the MUAS technique can be adapted for the study of epithelial ion transport in human endoscopic colonic specimens. The MUAS technique includes utilization of steady air suction in an easy manageable manner. The tissue is kept in place by air-suction making it unnecessary to surround the tissue with any kind of film (mounting the biopsies between polyesterfilms adds to the unstirred layer). The MUAS technique is fast, simple, easy to use, with minimal loss of tissue, associated with only minor degrees of edge damage, a high degree of viability, and reliable responses to various secretagogues and inhibitors for more than 2 hrs, and specimens can be easily changed for another specimen in the chamber setup. The mounting principle with air suction provides a stretch to the tissue, which ensures an optimal exposed area without causing damage to the tissue as evaluated by histology.

### Basal SCC and G

In the present study, basal electrical parameters varied over a wide range. Similar large variability has been noticed in other studies on human specimens from colon [8,20], table 1.

SCC and G were stable for more than 2 hrs, which is also consistent with another study using a different type of modified Ussing chamber [16]. Despite a subsequently progressive increase in SCC and G, reproducible responses could be obtained for more than 6 hrs to theophylline and for up to 4 hrs for 5-HT. This is somewhat surprising, but partially in agreement with a Ussing chamber study on human ileum, where transmucosal glucose fluxes were stable for 4 hrs despite progressive changes in SCC plus signs of epithelial histological changes after only 2 hrs [21].

In another study by Mullin and co-workers on histological normal surgical specimens from the left part of colon in patients with diverticular disease, SCC dropped initially from 250 to 50 μA·cm^2 ^and further down to 19 μA·cm^2 ^after 30 min – with large variations [22].

For the distal colon pronounced variations in resistance from 52 to 220 Ω·cm^2 ^has been reported [8]. Explanations for this large variability might be differences in the biopsy and mounting techniques, resulting in varying thickness of the samples and varying degrees of tissue damage, or that specimens are taken from different locations in the same region suggesting that true functional differences exist even within relative small distances in the intestine. Both these explanations can be corroborated by observations quoted in the literature [12,23,24]. Also the specimen size seems of importance since large-sized (50 – 70 mm^2^) surgical specimens exhibits higher resistance (31 – 312 Ω·cm^2^) [25-28] compared to small-sized (0.65 – 5 mm^2^) biopsy specimens (12 – 30 Ω·cm^2^) in this study and others [16,24,29].

Parallel changes in SCC and G was observed. With the (electronic) circuitry of the tissue, while measuring SCC across epithelia, a generator as the Na-pump is located in the basolateral membrane and the luminal membrane represents a *serial *conductance as does the internal conductance of the pump. Contrary, neighbor conductances to the pump in the basolateral membrane and conductances in paracellular pathways will act as *parallel *conductances. Hence, an increase in the luminal conductance will increase the SCC, while a change in parallel conductances will have no effect on measured transmural SCC.

Finally comparison of studies is hampered due to different levels in calcium concentrations in chamber bathing solutions varying from 0.5 – 3 mM, which is likely to be of importance as calcium regulates tight junction permeability [30].

### Possible effects of edge-damage

Edge-damage in the MUAS chamber have been discussed earlier and does not seem to be an explanation for the rather low resistances observed in the present study [9]. However, it should be recognized, that in case, as we did, a careful solution correction is performed immediately prior to tissue insertion, low tissue resistance as such does not affect the measured SCC as long as the transmural potential difference is zero. Correct short-circuiting eliminates shunt currents both in the tissue as well as through possible edge-damage pathways.

We conclude that colonic biopsies have much lower resistance than for instance surgical preparations for the Ussing chamber and in our study on colonic biopsies resistances are exceptionally low probably due to low calcium concentration in the media.

### Stimulation and inhibition experiments

The applied secretagogues and inhibitors all induced expected changes in SCC and was accompanied by changes in G.

For these effects it is assumed that, 5-HT activates various serotonergic receptors [31], carbachol muscarinic receptors [32], and forskolin adenylyl cyclase [33]. Theophylline inactivates phosphodiesterase [34], amiloride blocks electrogenic epithelial sodium channels [35], indometacin inhibits cyclooxygenase-enzymes [36], bumetanide inhibits sodium-potassium-chloride-co-transporter [37], while ouabain inhibits sodium-potassium pump [38].

The ionic basis for the observed changes in SCC and G were not investigated further, but are most likely due to opening and closing of ion channels and transporters for Cl^-^, HCO_3_^-^, Na^+ ^and K^+^, as demonstrated previously in various gut segments and species [39]. Another possibility for the observed SCC changes could be a depletion of salt or a build-up of an osmotic gradient in the lateral intercellular space with a closure or an opening of the space as seen for leaky epithelia [40]. However, these phenomena are unlikely during short circuit current voltage clamp to zero, which prevents the removal or built-up of salt in the paracellular pathway. Because we correct for the solution resistance just before every measurement, our tissues are well short circuited, and we can discard a closure of the intercellular space as an explanation for the observed fall in conductance.

### Drawback of the MUAS chamber technique

It is a drawback of the MUAS chamber technique that only 40% of biopsies can be used, based on the criteria of stable electrical parameters and expected responses to stimulators of ion secretion. Similarly the success-rate for duodenal specimens is reduced [9]. Reasons for the relatively low "success-rate" are still unclear. We have tried to change the experimental process in many ways (e.g. mechanical properties of the chamber and air-suction system, the bathing solutions, temperature etc.) without being able to improve the "success-rate". Some of the biopsies are too narrow to fit in the central opening of the discs and some can be mounted but demonstrate no response to stimulators or inhibitors. In some cases histology disclosed the likely cause due to substantial damage to the epithelium, which result from the biopsy forceps. Whether the success-rate using the MUAS chamber technique is different from other techniques for endoscopic biopsies cannot be assessed as such information in not yet available in the literature for other techniques.

## Conclusion

We conclude that epithelial ion transport is not significantly altered in diverticulosis and therefore the neuroepithelial integrity and function seems intact in patients with diverticulosis compared with controls. We also conclude that the MUAS chamber is adaptable for human colonic endoscopic biopsies for the study of epithelial ion transport.

## Abbreviations

SCC, Short Circuit Current;

G, Conductance;

MUAS, Modified Ussing Air-Suction;

5-HT, 5-hydroxytryptamine (serotonin);

D-patients, patients with diverticulosis;

t1/2, half time;

SEM, standard error of the mean;

## Competing interests

The author(s) declare that they have no competing interests.

## Authors' contributions

MCT and NK carried out the Ussing chamber studies and participated in the design of the study. PSO carried out the Ussing chamber studies and participated in the design of the study and performed the statistical analysis. SSP carried out the histoanatomy and helped to draft the manuscript. NB and MBH concieved of the study and helped to draft the manuscript. All authors read and approved the final manuscript.

## Pre-publication history

The pre-publication history for this paper can be accessed here:



## Supplementary Material

Additional file 1 SCC and G for controls and D-patients. The data provided represents a grouped vertical point plot of the distribution of SCC and G for the individual controls and D-patients. Click here for file
