# Supplementary figures and images for: Colonic epithelial ion transport is not affected in patients with diverticulosis
Source: BMC Gastroenterol. 2007 Sep 23;7:37. doi: 10.1186/1471-230X-7-37 (PMC2064914; doi:10.1186/1471-230X-7-37)

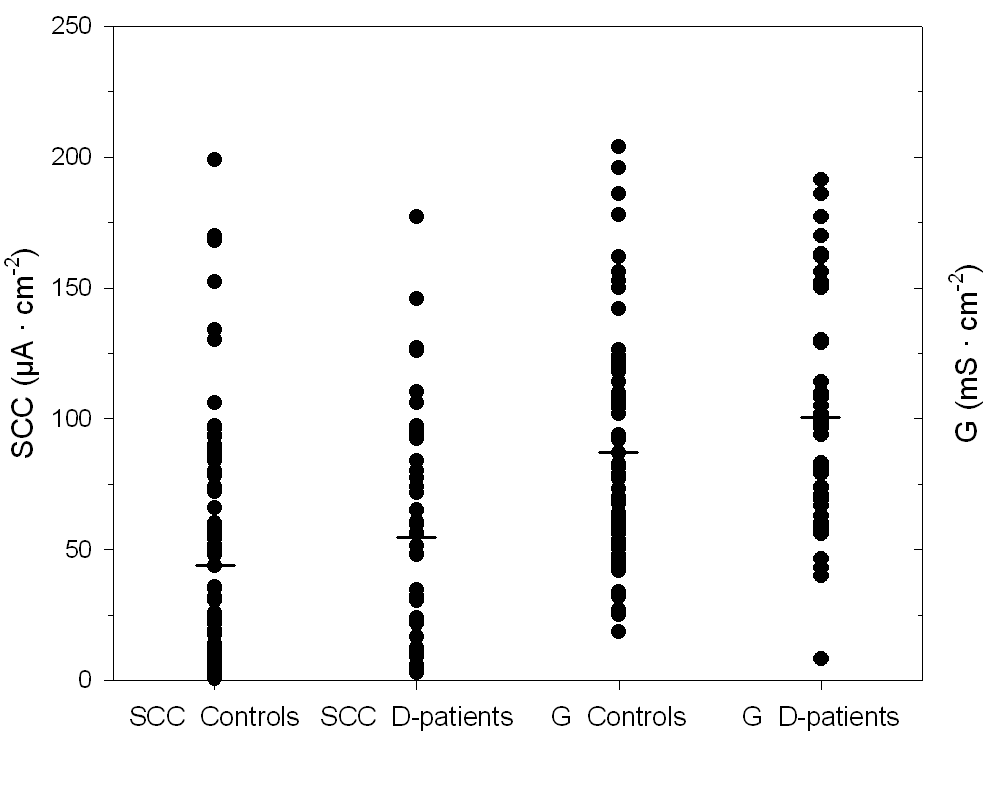

Supplement: Additional file 1 — SCC and G for controls and D-patients. The data provided represents a grouped vertical point plot of the distribution of SCC and G for the individual controls and D-patients. [file 1471-230X-7-37-S1.bmp]
